# Supplementary material for: Development of the social brain from age three to twelve years
Source: Nat Commun. 2018 Mar 12;9:1027. doi: 10.1038/s41467-018-03399-2 (PMC5847587; doi:10.1038/s41467-018-03399-2)
Supplement: Supplementary file 1 — Supplementary Information [file 41467_2018_3399_MOESM1_ESM.pdf]

Supplementary Figure 1

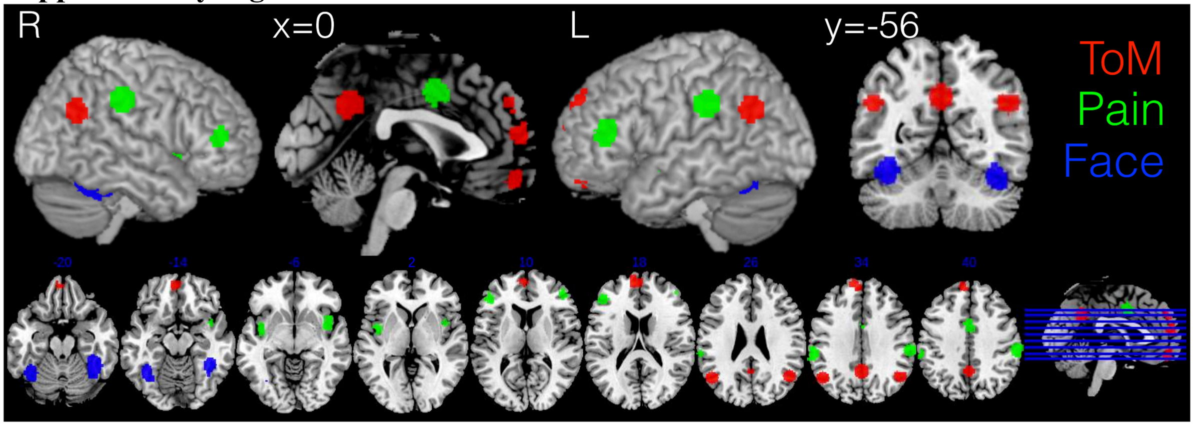

Supplementary Figure 1. Group Regions of Interest. ToM (red) and pain (green) regions were defined based on group-level contrast images in n=20 adults scanned by Evelina Fedorenko and colleagues (see Methods and Supplementary Figure 6). Bilateral fusiform regions (blue) were created by and described in[1]. See Supplementary Table 2 for ROI center coordinates and size.

Supplementary Figure 2

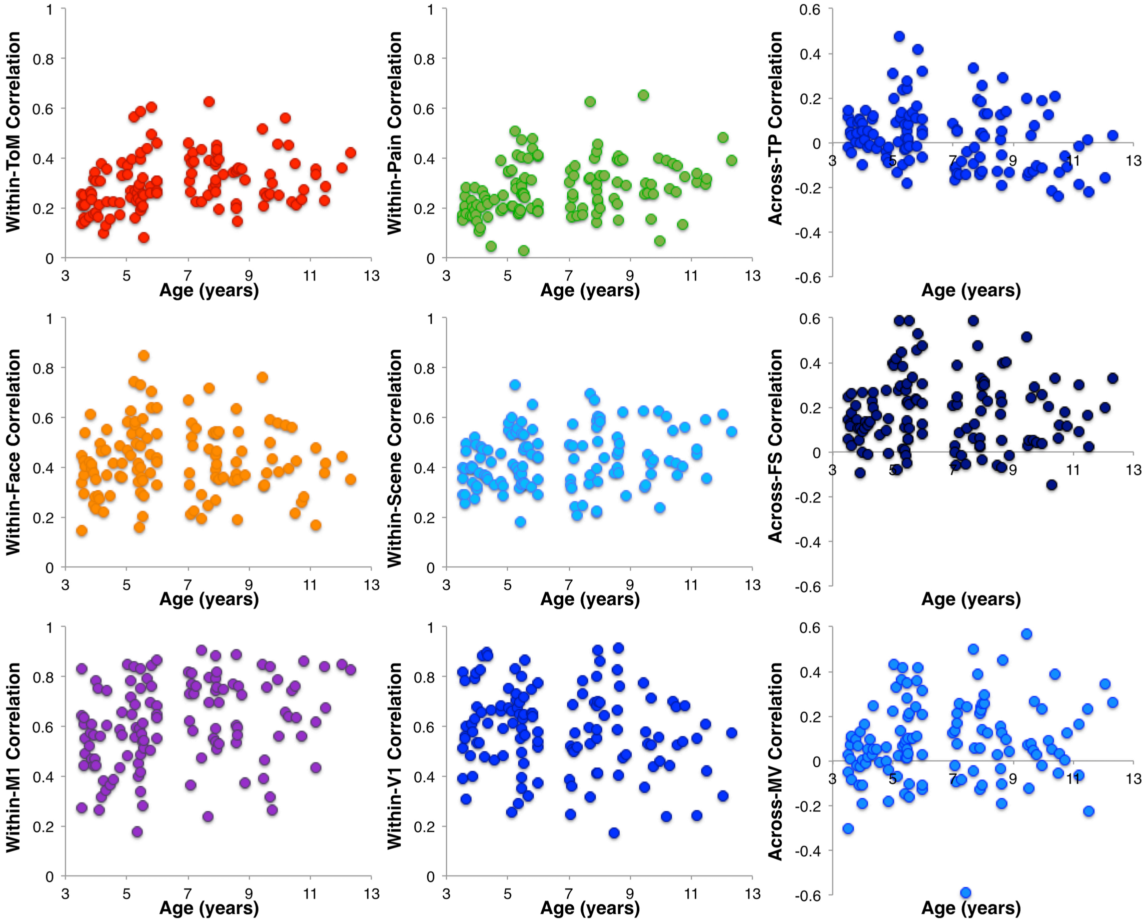

Supplementary Figure 2. Inter-regional correlations by age. Correlations are the raw (non z-scored) *r*-values (y-axis), calculated on the "raw" timecourses (without regression of the bilateral-M1 timecourse). Correlation values are shown for all children (n=122), with age on the x-axis. **Top row:** Within-ToM (red), Within-Pain (green), and across-ToM-Pain (dark blue) network correlations. **Middle row:** Within-Face (orange), Within-Scene (light blue), and across-Face-Scene (navy) network correlations. **Bottom row**: Within-M1 (purple), Within-V1 (bright blue), and across-M1-V1 (light blue) network correlations. See Results (main text) and Supplementary Figure 3 for statistics on change with age (all statistical tests used z-scored correlation values).

Supplementary Figure 3

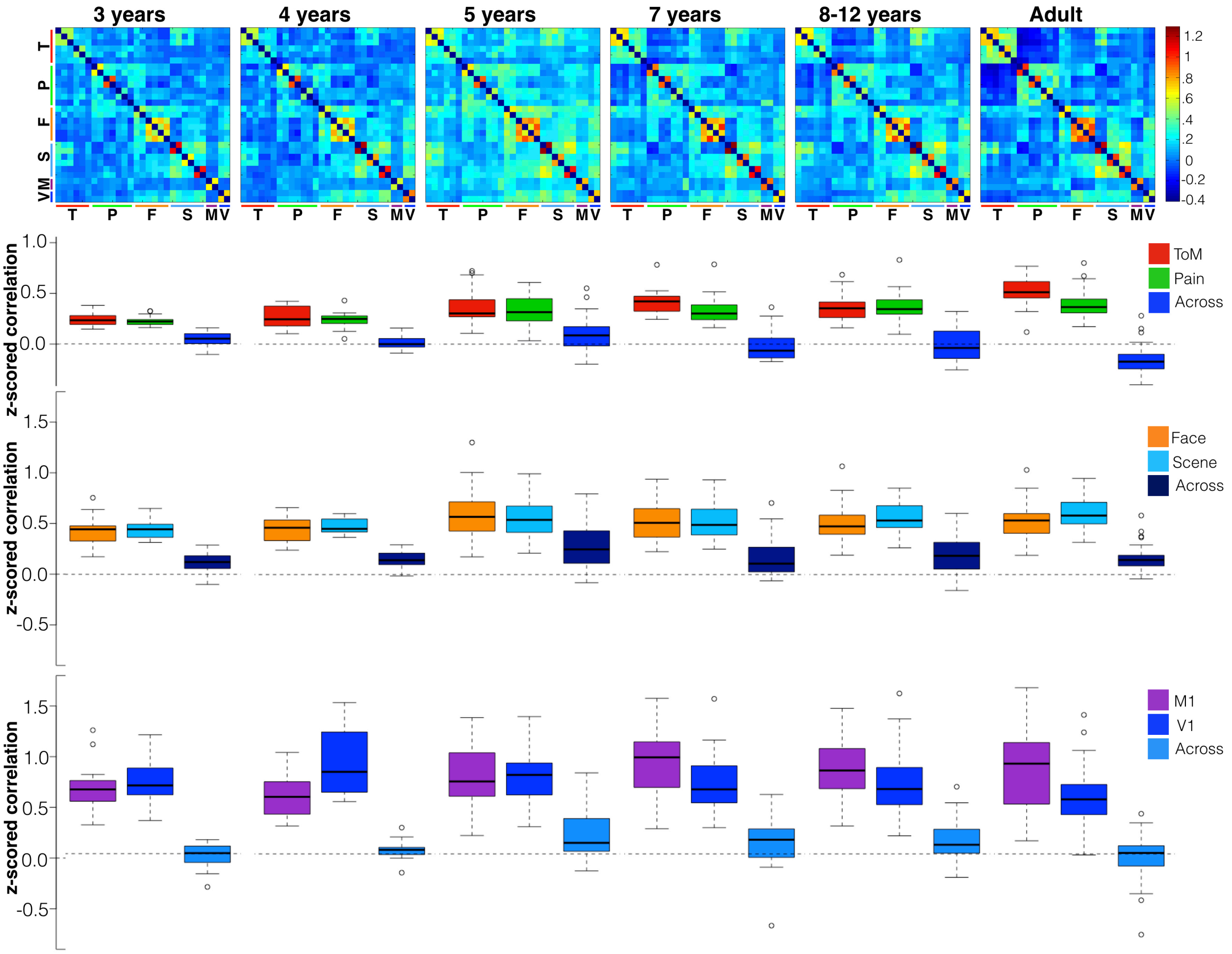

Supplementary Figure 3. Expanded inter-regional correlation analyses. Top row shows interregional z-scored correlation matrices for an expanded list of brain regions in the following order (ToM regions: RTPJ, LTPJ, PC, DMPFC, MMPFC, VMPFC, Pain regions: RS2, LS2, RInsula, LInsula, RMFG, LMFG, daMCC, Face regions: RSTS, LSTS, ROFA, LOFA, RFFA, LFFA; parcels from[1], Scene regions: RRSC, LRSC, RTOS, LTOS, RPPA, LPPA; parcels from[1], primary motor cortex: RPM, LPM, primary visual cortex: R Calcarine Sulcus, LCalcSulc. Primary motor and visual cortex ROIs are 10mm spheres drawn around peak coordinates generated with Neurosynth (http://neurosynth.org/; M1 coordinates: [38,-24,58], [-38,-20,58]; V1 coordinates: [-10 -86 2], [10 -86 2], see Methods). Boxplots show within- and across-network z-scored correlation values for all participants (n=122 children, n=33 adults), binned by age group, for ToM and Pain networks (top row of boxplots), Face and Scene networks (middle row) and bilateral primary motor and visual cortex regions (bottom row). All age correlation tests were spearman partial correlation tests, including amount of motion (number of artifact timepoints) as a covariate. Significant positive age correlations among children (n=122) are present for within-ToM (r_s=.39, p<.0001), within-Pain (r_s=.38, p<.0001), within-Scene (r_s=.23, p=.01), and within-M1 regions (r_s=.30, p=.001; within-Face: r_s=.06, p=.53, within-V1: r_s=-.16, p=.09). Because the within-M1 correlation increases with age, including it as a regressor in the interregional correlation analyses in the main text ensures that reported age effects in the ToM and pain networks are above and beyond developmental effects present in regions like primary motor

cortex. The M1 timecourse is not regressed out from the timecourses analyzed for this figure/the expanded IRC analysis. Across-ToM-Pain network correlations decrease with age (e.g., become more anti-correlated: r_s=-.26, p=.005). Across-Face-Scene and Across-M1-V1 correlations do not show significant change with age: Across-Face-Scene: r_s=.02, p=.8; Across-M1-V1: .18, p=.05). Positive correlations between within-ToM and within-Pain correlations and age were significantly stronger than within-Face and within-V1 correlations, but not significantly stronger than within-Scene and within-M1 correlations (Williams' test of differences in age correlations: Within-Face: vs. within-ToM: z=2.69, p=.01, vs. within-Pain: z=2.66, p=.01; within-Scene: vs. within-ToM: z=1.33, p=.18, vs. within-Pain: z=1.3, p=.19; M1: vs. within-ToM: z=.76, p=.44, vs. within-Pain: z=.73, p=.47; V1: vs. within-ToM: z=4.35, p=0 , vs. within-Pain: z=4.32, p=0). The across ToM-Pain anti-correlation was significantly stronger than the across Face-Scene and across M1-V1 anti-correlations (Face-Scene: z(122)=2.2, p=.03; M1-V1: z(122)=3.39, p=0). See Supplementary Figure 2 for scatter plots of raw correlation values by age, and Supplementary Table 4 for correlations between the average timecourse of each age group and adults, for these additional networks.

Supplementary Figure 4

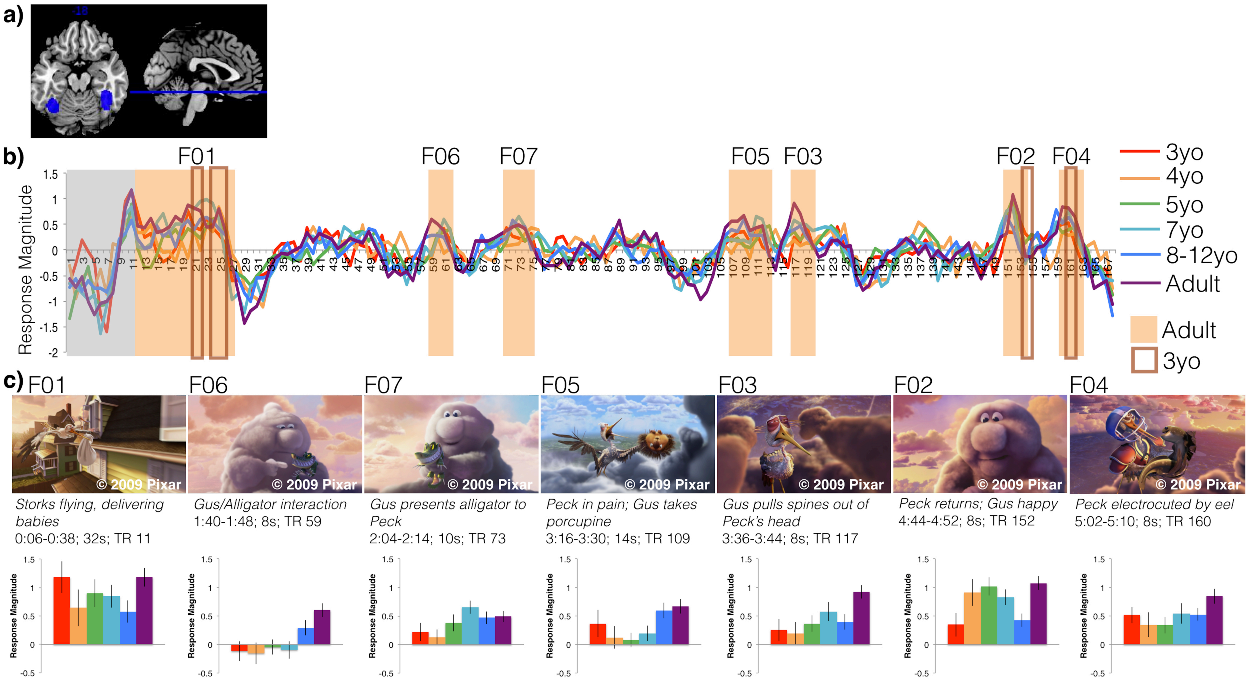

Supplementary Figure 4. Bilateral Fusiform Reverse Correlation Analysis. a) Bilateral fusiform regions of interest (ROIs). Regions are face parcels created with the group-constrained subject specific (GSS) method applied in n=30 adults, using a faces > objects contrast[1], and made publically available (http://web.mit.edu/bcs/nklab/GSS.shtml). A subset of participants (n=17 total; n=2 adults, n=12 8-12yos, n=1 7yo, 4yo, 3yo) had incomplete coverage of the ventral visual stream; however, all participants had measurable neural responses in at least 100 voxels in all face ROIs. **b)** Average timecourse of response extracted from bilateral fusiform ROIs, per age group. The average timecourse in bilateral fusiform ROIs in children was highly correlated with that of adults (pearson correlation: r=.86, $p<1.0x10^{-47}$). This correlation remained high when comparing adults to three-year-old children alone (pearson correlation: r=.72, $p<1.0x10^{-26}$). Shaded light orange blocks denote 7 events (88s total, M(SD) length 12.6(8.8)s) identified in a reverse correlation analysis of the timecourse of response in adult participants (n=33; see Methods); dark orange outlines denote 4 events (18s total M(SD) length 4.5(1)s) identified in a reverse correlation analysis of the timecourse of response in three-year-old children (n=17). Event labels (e.g. F01, F02) reflect rank order of magnitude of response in adults. A majority of the timepoints identified by the reverse correlation analysis in three year olds fall within adult events F02, F03, and F07 (8/9 TRs); the remaining timepoint immediately follows adult event F07. **c)** Example frame[2], short description, timing and duration, timepoint of peak response, and response magnitude by age group for each event identified in the reverse correlation analysis. Error bars represent standard error. Peak timepoints were chosen based on the adult data, included here for illustration. Statistical tests of age-related change were computed only on data from children (n=122). The magnitude of response in bilateral fusiform does not change with age among children (spearman partial correlation including motion as covariate; Bonferroni correction for multiple comparisons α =.0071, correcting for 7 events/tests; $|r|$s<.24, ps>.01). All events included at least one face, and close-ups of faces; some events featured particularly salient faces (e.g. faces with painful expressions: F01, F02, F05). Images ©2009 Pixar, reused with permission. These images are not covered under the CC BY license for this article.

Supplementary Figure 5

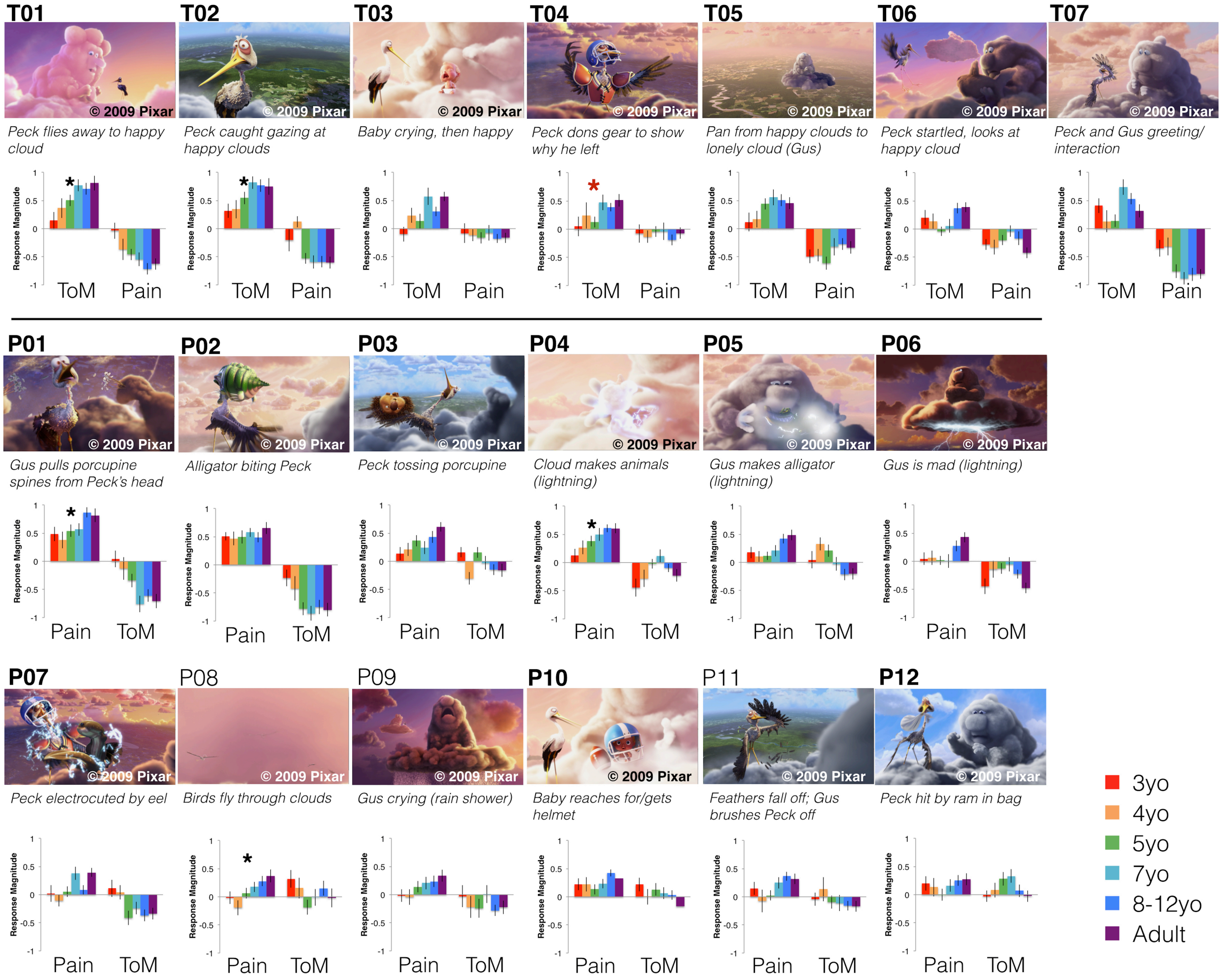

Supplementary Figure 5. Reverse Correlation Analysis: ToM and Pain events. The reverse correlation analysis in adults identified seven ToM events (top) and twelve pain events (bottom). For each event, an example frame and description are given[2]. The bar graphs show the average response magnitude of response per age group in the ToM and Pain networks, for each event. Peak timepoints were chosen based on the adult data, included here for illustration. Statistical tests of age-related change were computed only on data from children (n=122). Asterisks denote events that evoke significantly greater responses with age (black; partial spearman correlation controlling for motion and correcting for multiple comparisons (MC) (19 events, α =.0026)), or ToM behavioral performance (red; linear regression including age and motion as additional predictors, and correcting for MC (7 events, α =.007)). Event labels (e.g. T01, T02) in bold type are those that were replicated in an independent sample of adults (n=20; Supplementary Figure 6). See Supplementary Table 3 for event timing, duration, and full descriptions.

Images ©2009 Pixar, reused with permission. These images are not covered under the CC BY license for this article.

Supplementary Figure 6

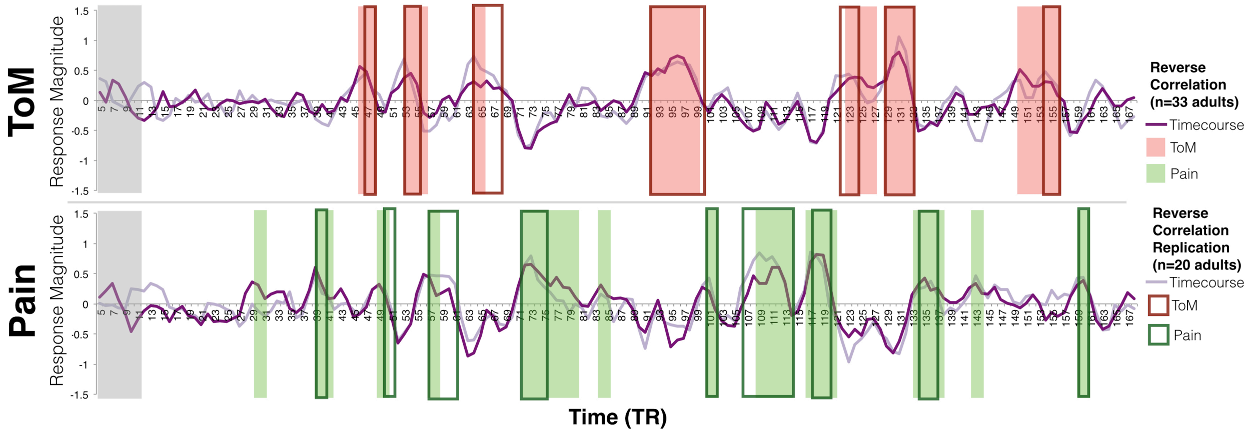

Supplementary Figure 6. Comparison of reverse correlation analysis results across two adult samples. We analyzed fMRI data from an independent sample of adults (n=20), collected by Evelina Fedorenko's lab, who viewed "Partly Cloudy" [2] in the scanner. We used this sample to create independent, stimulus-tailored group ROIs (see Methods). We also used this sample to test whether the events identified by the reverse correlation analysis in our adult sample (n=33) were replicated in an independent sample of adults. Because we used this independent sample of adults to create the group ROIs used for our sample of interest, we created a different set of group ROIs for the reverse correlation replication analysis of these participants (to avoid using non-independent ROIs). The group ROIs used in the independent sample were 10mm spheres surrounding peak coordinates reported in previous publications (ToM regions[3]; Pain matrix[4]). This figure shows the average timecourse of response in the primary adult sample (n=33, dark purple) and the independent replication sample (n=20, light purple), in each network. Shaded blocks indicate events identified by reverse-correlation in the primary adult sample; dark borders indicate events identified by reverse-correlation in the replication sample (ToM: red, Pain: green). Seven ToM and nine pain events were identified in the reverse correlation analysis of this independent sample of adults (ToM: 60s total, M(SD) length: 8.6(4.6)s, Pain: 66s total, M(SD) length: 7.3(4.4)s). All events identified in the independent group of adults (using group ROIs[3,4]) were also identified in our adult participants. Three pain events that were identified in our primary sample of adults were not labeled as events in the independent sample of adults: P08, P09, and P11 (9/12 overlapping pain events comprised of 52s of overlap, and 7/7 overlapping ToM events comprised of 54s of overlap). Thus, the reverse correlation analysis approach successfully identifies events that reliably evoke responses in ToM and pain brain regions across adult subjects, and across two independent adult samples. This suggests that this approach is particularly well suited for identifying events for further analyses of changes in neural responses with development.

Supplementary Figure 7

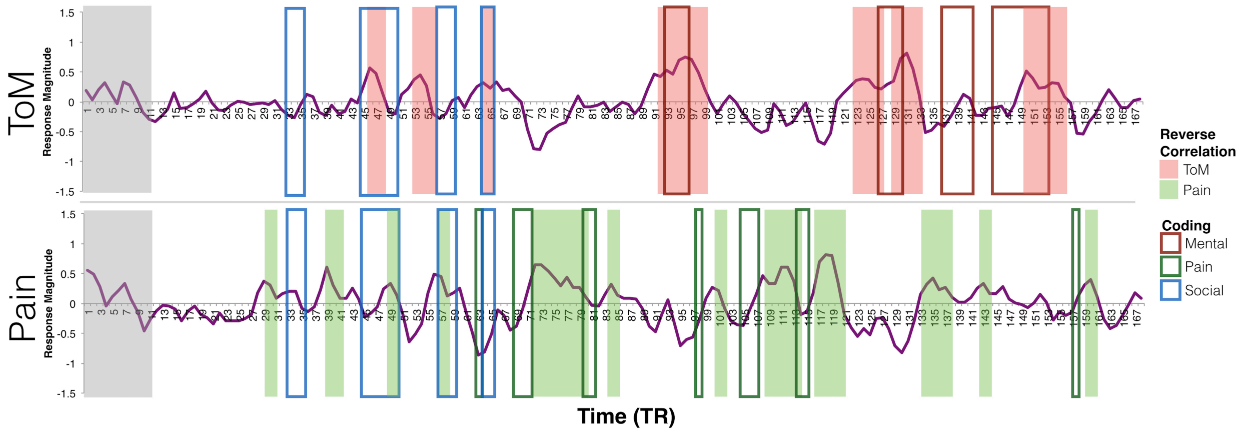

Supplementary Figure 7. Comparison of Reverse Correlation analysis and original event coding. A previous study coded "mental," "social," and "pain" events of the movie stimulus, in order to compare the magnitude of response across conditions and localize ToM and pain brain regions using contrasts (ToM regions: Mental > Pain; Pain matrix: Pain > Mental)[5]. We compared the coding created by the experimenters to the event labels suggested by the reverse correlation analysis in our adult participants (n=33). This figure shows the average timecourse of response in adult participants in ToM (top) and pain (bottom) networks. Shaded blocks indicate events identified by reverse correlation analysis (ToM: red, Pain: green). Colored borders indicate condition labels constructed by previous experimenters for the purpose of using the movie stimulus as a functional localizer for identifying ToM and pain brain regions[5]. While most ToM events identified by the reverse correlation analysis were at least partially included in the original coding (6/7 ToM events labeled as Mental or Social), only two of twelve pain events were included in the coding. This lends support to the use of reverse correlation analysis for identifying reliable events that evoke responses in particular regions, rather than experimenter-based coding, for further study. The reverse correlation analysis approach may be useful for refining theories about the function of these networks of brain regions.

Supplementary Figure 8

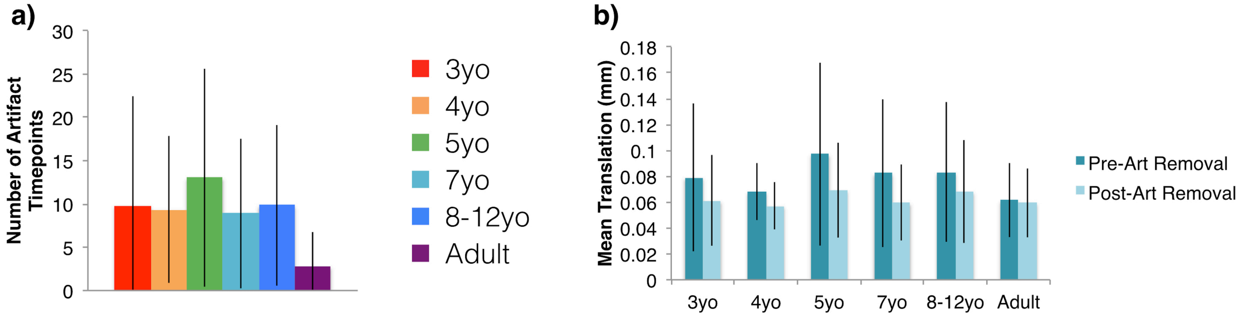

Supplementary Figure 8. Amount of motion in fMRI data. a) Number of artifact timepoints identified in the timecourse of response (one run per participant, 168 timepoints total), by age group (3yo: n=17, 4yo: n=14, 5yo: n=34, 7yo: n=23, 8-12yo: n=34, adult: n=33). Artifact timepoints are timepoints in which there is 2mm motion and/or a global signal change greater than three standard deviations from the mean, relative to the previous timepoint. Error bars show standard deviation from the mean. **b)** Mean translation (motion in x, y, z directions) in millimeters per age group, including (dark) and excluding (light) artifact timepoints. Error bars show standard deviation from the mean.

Supplementary Table 1

Age Group	N	Age Range M (SD)	Gender (#F)	Handedness (R/L/Ambi)	Raw IQ M (SD)	Scaled/ Standard IQ M (SD)	DCCS Summary M (SD)	ToM Score M (SD)	Explicit FB Groups (P/I/F)
**3yo**	17	3.52-3.99<br>3.75 (.18)	10	15/2/0	15.5 (3.4)	10.4 (2.3)	1.75 (.93)	.54 (.18)	4/4/9
**4yo**	14	4.06-4.86<br>4.43 (.29)	8	13/0/1	16.9 (3.9)	9.64 (3.4)	2.29 (.61)	.63 (.15)	3/7/4
**5yo**	34	5.01-5.99<br>5.51 (.29)	16	26/6/2	20.3 (5.2)	111.7 (13.3)	2.32 (.47)	.73 (.14)	23/9/2
**7yo**	23	7-7.96<br>7.54 (.37)	11	23/0/0	29.4 (6.8)	116.7 (16.8)	NA	.88 (.09)	20/3/0
**8-12yo**	34	8-12.3<br>9.77 (1.18)	19	33/1/0	35.6 (3.9)	120 (11.7)	NA	.96 (.06)	34/0/0
**Adult**	33	18-39<br>24.8 (5.3)	20	32/1/0	NA	NA	NA	NA	NA

Supplementary Table 1. Demographic information and behavioral data by age group. Number of participants (N), age range and average and standard deviation of age (years), gender, handedness, raw and standardized measures of nonverbal IQ, DCCS summary score (possible range: 0-3)[6], ToM score (proportion of all questions answered correctly; possible range: 0-1), and number of children in each explicit false belief task group (pass, inconsistent, fail), per age group. Nonverbal IQ was measured via the WPPSI block design task for children ages 3-4 years[7], and via the KBIT-2 matrices task for children ages 5-12 years[8]. Children ages 7 and older did not complete the DCCS task. False belief task passers answered 5 or 6 of 6 questions correctly, inconsistent performers answered 3-4 questions correctly, failers answered at most 2 of 6 questions correctly.

Supplementary Table 2

**Network**<br>Contrast	**ROI**	**Center Coordinate**	**Size (voxels)**
**ToM**			
ToM > Pain	RTPJ	[48 -60 30]	376
	LTPJ	[-48 -62 30]	368
	PC	[0 -54 34]	382
	DMPFC	[-6 54 36]	217
	MMPFC	[-4 58 16]	275
	VMPFC	[-4 56 -16]	198
**Pain**			
Pain > ToM	RS2	[60 -28 38]	368
	LS2	[-62 -32 34]	269
	Rinsula	[42 6 -6]	309
	Linsula	[-42 -2 -4]	240
	RMFG	[50 42 12]	142
	LMFG	[-46 36 14]	256
	AMCC	[0 2 42]	249
**Face**			
Face > Object	RFFA	[38 -42 -22]	1019
	LFFA	[-40 -52 -18]	531

Supplementary Table 2. Group regions of interest. Contrast used, regions identified, peak/center coordinate [x y z], and size (number of voxels) for each region of interest in the ToM network and Pain matrix, and for the bilateral fusiform regions used in Supplementary Note 1 and Supplementary Figure 4. See Supplementary Figure 1 for a visualization of these regions of interest.

Supplementary Table 3

	Event	Time	Duration (s)	Peak Timepoint (TR)	Description
**ToM Events**	T01*	4:00-4:10	10	131	Peck flies away from Gus after seeing the baby shark (T06), landing on another (happier) cloud. Peck and the happy cloud seemingly laugh together about Gus.
	T02*	2:46-3:02	16	96	Peck stares longingly at a happy cloud who is making puppies. Gus notices this, and looks worried. Peck notices that Gus caught him looking longingly, and feels bashful.
	T03*	1:14-1:20	6	46	Baby crying, then becomes happy when given a helmet.
	T04*	4:42-4:56	14	150	Peck dons football gear, to explain to Gus that he did not abandon him, but rather was acquiring protective equipment such that he could continue to deliver Gus's (dangerous) babies.
	T05*	1:28-1:36	8	54	Pan from happy clouds to Gus, who expresses loneliness.
	T06*	3:48-3:58	10	124	Peck is startled by the baby shark Gus has made. He notices a happy cloud who is making chicks.
	T07*	1:50-1:54	4	64	Peck and Gus greet each other happily (they are friends).
**Pain Events**	P01*	3:36-3:46	10	118	Gus pulls porcupine spines out of Peck's head.
	P02*	2:06-2:24	18	73	Alligator baby is biting Peck's head repeatedly.
	P03*	3:20-3:32	12	111	Peck tosses porcupine baby; expressing pain.
	P04*	1:00-1:06	6	39	Cloud makes baby animals (lightning).
	P05*	1:34-1:40	6	56	Gus makes baby alligator (lightning).
	P06*	4:10-4:20	10	135	Gus expresses anger (lightning).
	P07*	5:02-5:06	4	160	Peck is electrocuted by baby eel (lightning).
	P08	0:42-0:46	4	29	Flock of birds fly through clouds.
	P09	4:28-4:32	4	143	Gus begins to cry heavily (rain shower).
	P10*	1:20-1:24	4	49	Baby reaches for and is given helmet.
	P11	2:30-2:34	4	84	Peck's feathers fall off; Gus brushes Peck off.
	P12*	3:04-3:08	4	100	Peck is hit by baby ram in bundle he is trying to carry.

**Indicates that event was replicated in reverse correlation analysis of an independent sample of adults (see Figure S6)*

Supplementary Table 3. ToM and Pain Event details. Time (in stimulus), duration (seconds), and peak timepoint (TR) and description for each ToM and Pain event.[2] Peak timepoint is the timepoint with the greatest average response magnitude in adult participants. Event labels (T01, P01) reflect rank order of average response magnitude in adults. *Asterisks indicate events replicated in reverse correlation analysis of an independent sample of adults (Supplementary Figure 6).

Supplementary Table 4

Age Group	ToM	Pain	Face	Scene	M1	V1	Bi-FFA
**3yo**	0.28	0.60	0.75	0.61	0.11	0.53	0.72
**4yo**	0.31	0.56	0.59	0.67	0.06	0.61	0.60
**5yo**	0.60	0.73	0.71	0.77	0.35	0.78	0.68
**7yo**	0.72	0.83	0.84	0.80	0.44	0.74	0.82
**8-12yo**	0.82	0.89	0.86	0.85	0.53	0.76	0.83

Supplementary Table 4. Average timecourse correlations. This table provides the Pearson correlation value (*r*) between the average timecourse of response in each network included in the expanded IRC analysis, for each age group, and the corresponding average timecourse of response in adults. These timecourses are the same as those used for the reverse correlation analysis (the M1 timecourse is not included as a regressor), prior to z-normalization. All correlations are significantly positive (ps<.0005) except those shaded in grey (3yo M1: p=.16; 4yo M1: p=.46).

Supplementary Note 1: Face events overlap analysis

We conducted an overlap analysis to determine whether the amount of overlap between timepoints identified as face events and timepoints identified as ToM or pain events was significantly different from that expected by chance. The overlap analysis was identical to the analysis used to determine whether ToM and pain events were significantly non-overlapping, described in Methods. The permuted face timecourses included seven face events, with durations of 16, 4, 5, 7, 4, 4, 4 TRs (see Supplementary Figure 4). Face and ToM events had 4 TRs of overlap in the actual timecourses; 111/1000 random permutation tests showed the same or smaller amount of overlap ($p=.11$). Face and pain events had 15 TRs of overlap in the actual timecourses; 928/1000 random permutation tests showed the same or smaller amount of overlap ($p=.93$). Thus, the amount of overlap between face and ToM events, and face and pain events, did not differ from that expected by chance.

Supplementary References

1. Julian, J. B., Fedorenko, E., Webster, J. & Kanwisher, N. An algorithmic method for functionally defining regions of interest in the ventral visual pathway. *NeuroImage* **60,** 2357–2364 (2012).
2. Reher, K. (Producer), & Sohn, P. (Director). *Partly Cloudy* [Motion Picture]. United States: Pixar Animation Studios and Walt Disney Pictures (2009).
3. Dufour, N., Redcay, E., Young, L., Mavros, P.L., Moran, J.M., Triantafyllou, C., Gabrieli, J, Saxe, R.. Similar Brain Activation during False Belief Tasks in a Large Sample of Adults with and without Autism. *PLoS ONE* **8,** e75468 (2013).
4. Bruneau, E. G., Jacoby, N. & Saxe, R. Empathic control through coordinated interaction of amygdala, theory of mind and extended pain matrix brain regions. *NeuroImage* **114,** 105–119 (2015).
5. Jacoby, N., Bruneau, E., Koster-Hale, J. & Saxe, R. Localizing Pain Matrix and Theory of Mind networks with both verbal and non-verbal stimuli. *NeuroImage* **126,** 39–48 (2016).
6. Zelazo, P. D. The Dimensional Change Card Sort (DCCS): a method of assessing executive function in children. *Nat Protoc* **1,** 297–301 (2006).
7. Wechsler, D. Manual for the WPPSI-R. *New York: The Psychological Co* (1989).
8. Kaufman, A. S. KBIT-2: Kaufman Brief Intelligence Test. Minneapolis, MN: NCS Pearson. (1997).
